# Association of Frailty Index with Clinical BPH Progression and Serious Adverse Events: the MTOPS Trial

**DOI:** 10.1093/geroni/igab046.2992

**Published:** 2021-12-17

**Authors:** Scott Bauer, Kristine Ensrud, Louise C Walter, Anne M Suskind, William A Ricke, Teresa T Liu, Kevin T McVary, Kenneth Covinsky

**Affiliations:** 1 UCSF and San Francisco VA, San Francisco, California, United States; 2 University of Minnesota, Minneapolis, Minnesota, United States; 3 University of California, San Francisco, San Francisco, California, United States; 4 University of Wisconsin School of Medicine and Public Health, Madison, Wisconsin, United States; 5 Stritch School of Medicine and Loyola University Medical Center, Maywood, Illinois, United States

## Abstract

Lower urinary tract symptoms due to suspected benign prostatic hyperplasia (BPH) are increasingly treated with medications targeting obstruction among older men, but frailty may represent a novel risk factor for this condition. Our objective was to assess the associations between frailty and clinical BPH progression or serious adverse events (SAE) among 3047 men, age 50-89 years, enrolled in the Medical Therapy of Prostatic Symptoms Study, a placebo-controlled RCT of doxazosin, finasteride, or combination therapy on clinical BPH progression. We created a frailty index using 69 items collected at baseline and categorized men as fit (0-0.1), less fit (0.1-<0.25), or frail (0.25-1.0). The primary outcomes were time to 1) first composite event of clinical BPH progression, and 2) SAE requiring hospitalization. Cox proportional hazards models were adjusted for demographics, intervention, BPH surrogates, and comorbidities. At baseline, 28% men were fit, 58% were less fit, and 14% were frail. During follow-up (mean 4.5 years), the incidence rate of clinical BPH progression was 2.2/100p-y among fit, 3.0/100p-y among less fit (HR =1.28, 95% CI 0.98, 1.67), and 4.1/100p-y among frail men (HR=1.60, 95% CI 1.13, 2.26). Among men randomized to combination therapy, the SAE incidence rate was 3.4/100p-y for fit men versus 12.7/100p-y for frail men (HR=5.98, 95% CI 3.76, 9.52). In conclusion, frailty is independently associated with greater risk of both clinical BPH progression and SAE. The decision to initiate medical therapy for BPH among frail men should therefore include a discussion of both benefits and risks via shared decision making.

